# Carbonic anhydrase IX (CA-IX) and high-risk human papillomavirus (H-HPV) as diagnostic biomarkers of cervical dysplasia/neoplasia in Japanese women with a cytologic diagnosis of atypical glandular cells (AGC): a Gynecologic Oncology Group (GOG) Study

**DOI:** 10.1038/sj.bjc.6606049

**Published:** 2010-12-14

**Authors:** S-Y Liao, W H Rodgers, J Kauderer, T A Bonfiglio, K M Darcy, R Carter, L Levine, N M Spirtos, N Susumu, K Fujiwara, J L Walker, M Hatae, E J Stanbridge

**Affiliations:** 1 Department of Epidemiology, School of Medicine, University of California at Irvine, Irvine, CA 92868, USA; 2 Department of Pathology, Lenox Hill Hospital, New York, NY 10021, USA; 3 GOG Statistical and Data Center; Roswell Park Cancer Institute, Buffalo, NY 14263, USA; 4 Department of Pathology, University of Rochester Medical Center, Rochester, NY 14642, USA; 5 Department of Obstetrics and Gynecology, University of Texas, Galveston, TX 77555, USA; 6 Division of Gynecologic Oncology, University of Nevada School of Medicine and Women’s Cancer Center, Las Vegas, NV 89109, USA; 7 Department of Obstetrics and Gynecology Keio University School of Medicine, Tokyo 160 8582, Japan; 8 Department of Gynecologic Oncology, Saitama Medical University International Medical Center Hidaka-City, Saitama 350 1298, Japan; 9 Department of Obstetrics and Gynecology, University of Oklahoma Health, Sciences Center, Oklahoma City, OK 73190, USA; 10 Department of Microbiology & Molecular Genetics, School of Medicine, University of California at Irvine, Irvine, CA 92868, USA

**Keywords:** CA-IX, H-HPV, AGC diagnosis, cervix

## Abstract

**Background:**

High-risk human papillomavirus (H-HPV) infection is linked to cervical neoplasia but its role in detecting cervical glandular lesions (GLs) is unclear. Carbonic anhydrase IX (CA-IX) is a hypoxic biomarker that is highly expressed in neoplastic cervical GLs. The diagnostic utility of these biomarkers was evaluated by the Gynecologic Oncology Group in Japanese women with a cytological diagnosis of atypical glandular cells.

**Methods:**

Immunostaining was used to detect CA-IX in a conventional Pap smear. Immunoreactivity of CA-IX was interpreted by a panel of pathologists blinded to the histological diagnosis. Polymerase chain reaction was used to detect H-HPV in a liquid-based cytology specimen.

**Results:**

Significant cervical lesions (SCLs), defined as cervical intraepithelial neoplasia (CIN2, CIN3), adenocarcinoma *in situ* or invasive carcinoma, were observed in 37/88 (42%) of women. CA-IX testing alone (*n*=88) had a sensitivity of 89, 100 or 73% for SCLs, GLs or significant squamous lesions (SLs), respectively, with a false negative rate (FNR) of 14%. Testing for H-HPV (*n*=84) had a sensitivity of 65, 53 or 80% for SCLs, GLs or SLs, respectively, with a FNR of 22%. The combination of CA-IX and H-HPV testing had a sensitivity of 97, 100 or 93% for SCLs, GLs or SLs, respectively, with a FNR of 5%. Among eight H-HPV-negative GLs, six (75%) had a diagnosis of lobular endocervical glandular hyperplasia (LEGH).

**Conclusion:**

The combination of CA-IX and HPV testing improved the diagnostic accuracy. The low rate of H-HPV positivity in the GLs was associated with coexisting LEGH independent of H-HPV.

Cervical cancer is the second most common cause of cancer death in women worldwide, with an estimated 510 000 newly diagnosed cervical cancer cases and 288 000 deaths annually (Pagliusi, 2006). Although implementation of nationwide cervical cytological screening programs has led to a dramatic decline in the incidence of cervical squamous cell carcinoma, the rate of endocervical adenocarcinomas is on the rise, particularly in young women (Smith *et al*, 2000; Bray *et al*, 2005). The false negative rate in the cytological test for cervical adenocarcinomas, generally speaking, is higher than that for squamous lesions (SLs) (Makino *et al*, 1995). Several factors may contribute to such a difference; ill-defined cytological criteria for separating neoplastic glandular cells from benign mimics may have an important role.

In 2001, the Bethesda System introduced the term ‘atypical glandular cells (AGCs)’, replacing the term ‘AGCs of undetermined significance’, thereby classifying glandular cell abnormalities exceeding those typical reactive changes, but lacking features diagnostic of adenocarcinoma, into the following three categories: AGC of unclear cell origin, atypical endocervical cells and atypical endometrial cells (Solomon *et al*, 2002). However, in clinical practice, such a sub-categorization of AGC remains a diagnostic challenge with poor inter-observer agreement. The studies show that 17–80% (mean, 41%) of women with an AGC diagnosis were found to harbour significant cervical lesions (SCLs), including high-grade cervical intraepithelial neoplasia (CIN2 and CIN3), adenocarcinoma *in situ* (AIS) and invasive carcinoma. The rate of invasive carcinoma has been reported to be up to 10% (Liao and Stanbridge, 2000; Lee *et al*, 2002; Cangiarella and Chhieng, 2003).

From the clinical point of view, young women with AGC are often treated aggressively with cervical conization because of the relative lack of accuracy of colposcopy and endocervical curettage for excluding SCLs. It has been well documented that SCLs, including invasive carcinoma, may exist in AGC patients even when the results of colposcopic examination and endocervical sampling are normal (Andersen and Arffmann, 1989). Therefore, from a cost-benefit standpoint, and from a desire to avoid unnecessary invasive procedures, an accurate screening method or test is needed to determine which women with a cytological diagnosis of AGC harbour a SCL. In the recent years, many biomarkers have been developed and, among those, human papillomavirus (HPV) and carbonic anhydrase IX (CA-IX) appear to be particularly promising.

Infection with oncogenic high-risk HPV (H-HPV) strain(s) is widely accepted to be an important aetiologic factor for cervical cancer (zur Hausen, 2002; Schiffman *et al*, 2007; Bosch *et al*, 2008). Clinical trials have established the importance of H-HPV testing for the detection of significant SLs, including CIN2, CIN3 and squamous cell carcinoma (Solomon *et al*, 2001). Furthermore, H-HPV has been detected in 80–90% of adenocarcinomas and their precursor glandular lesions (GL) (Pirog *et al*, 2000; Bosch *et al*, 2008). However, there are only limited data available with respect to H-HPV testing as a diagnostic tool in the detection of glandular neoplasia.

In the 1990s, the antigen MN was identified (Zavada *et al*, 1993). The antigen MN is a transmembrane glycoprotein, and is a member of the carbonic anhydrase gene family, and is more specifically designated carbonic anhydrase IX (CA-IX) (Opavsky *et al*, 1996). The CA-IX is a biomarker of several human tumours, including carcinomas of the cervix and kidney (Liao *et al*, 1994, 1997). The expression of CA-IX in cancerous tissues, and its absence in normal counterparts, has led to the speculation that it has a function in carcinogenesis (Wykoff *et al*, 2000; Ivanov *et al*, 2001). Its expression is controlled by the transcription factor, hypoxia-inducible factor-1, and is upregulated in hypoxic regions of tumour tissues (Swietach *et al*, 2007).

In a survey of benign and neoplastic cervical tissues and Pap smears (PSs), it was observed that virtually all AGC associated with AIS and adenocarcinoma expressed high levels of CA-IX antigen, but this biomarker was rarely detected in the benign cervical cells/cervical tissues. This suggested the possibility that the expression of CA-IX may serve as a useful biomarker for diagnosing AIS and adenocarcinoma (Liao *et al*, 1994, 2009; Liao and Stanbridge, 1996, 2000).

In 1998, the Gynecologic Oncology Group (GOG), a national multi-institutional clinical trials group, supported by the US National Cancer Institute, conducted a study of women with a cytological diagnosis of AGC. In total, 25 institutions in the United States and 11 institutions in Japan participated in the trial. Here, we report the results from the Japanese cohort. The objective of this study, included in GOG protocol #171, was to determine whether CA-IX expression in a conventional PS is a diagnostic biomarker for a SCL in Japanese women with a cytological diagnosis of AGC and to explore the diagnostic value of H-HPV testing alone or in combination with CA-IX.

## Materials and methods

GOG protocol #171 initiated accrual in the United States in 1998 and in Japan in 2003. The criteria of AGC diagnosis for patient enrollment were based on the 1991 Bethesda System classification and conventional PSs were used. PCR was used to detect H-HPV DNA in corresponding liquid-based cytology specimens. The protocol closed for accrual in both countries in 2005.

### Eligibility and clinical management

Women over the age of 18 years, with a referring diagnosis of AGC, who were expected, on a clinical basis, to undergo complete histological evaluation of the cervical transformation zone within 6 months of the initial cytological diagnosis were enrolled. Patients with a history of endometrial hyperplasia and/or carcinoma of the uterine corpus, cervix and vagina; previous or concurrent chemotherapy and/or radiation to the uterine corpus, cervix and vagina; or HIV infection were excluded. All patients received colposcopic examination, cervical biopsy, endocervical curettage and/or an endometrial biopsy as clinically indicated, as well as either loop electrosurgical excision cone biopsy of the cervix with an endocervical curettage, a cold knife cone biopsy of the cervix with/without an endocervical curettage or a hysterectomy within 6 months of the initial cytological diagnosis of AGC. An endometrial biopsy or curettage was obtained in all premenopausal and postmenopausal women, as well as in all patients with a negative cone biopsy of the cervix. Patients with a negative diagnosis after the cervical cone biopsy, but not undergoing a hysterectomy, were to be followed with routine PS screening every 6 months for 2 years. Informed consent consistent with federal, state and local requirements was obtained before enrollment. Before activation, the protocol was approved by the National Cancer Institute, Division of Cancer Prevention and the GOG Human Research Committee, and annually by the Institutional Review Board at each of the participating institutions.

### Sample collection

After enrollment, two cytology specimens were collected before surgical procedures were performed. At first, a spray-fixed conventional study PS was taken with a spatula and cytobrush and stained according to the Pap method. A second sample was obtained with a sampling device and suspended in 20 ml of the PreservCyt Solution (Cytyc/Hologic, Marlborough, MA, USA) for H-HPV DNA testing.

Histological samples were taken by punch biopsy, endocervical and/or endometrial curettage or loop electrosurgical excision or cold knife cone excision at colposcopy and/or hysterectomy, as clinically indicated. Specimens were fixed in 10% formalin, paraffin embedded, sectioned and stained with hematoxylin and eosin.

### Histological diagnosis

Hematoxylin and eosin-stained slides of the most abnormal lesions from each diagnostic procedure were reviewed centrally by teams of two pathologists from the GOG Pathology Committee who reached a consensus diagnosis. Disparities were arbitrated by a third GOG pathologist. A positive diagnosis, coded as a SCL, reflects the presence of CIN2, CIN3, AIS or invasive carcinoma in the cervix. A negative diagnosis represents the absence of SCLs, including CIN1 and atypia. Atypia was defined as glandular and SLs in which cellular atypia falls short of AIS and CIN1. Evaluation of the entire cervical transformation zone was required to make a negative diagnosis, but not to make a positive diagnosis. The significant lesions were restricted to the cervix and there was no case of vaginal dysplasia/neoplasia without the coexisting cervical lesions or lesions outside the uterus identified in the study. Retrospective evaluation of the histology in all cases with the knowledge of the H-HPV and CA-IX status was conducted by one pathologist (SYL). The hematoxylin and eosin-stained sections of all GLs, and those cases that were negative for H-HPV DNA, but expressed CA-IX, were also independently reviewed by a second pathologist (WHR) who was blinded to the clinical data.

### Detection of CA-IX in a conventional study PS

**Figure 1:**
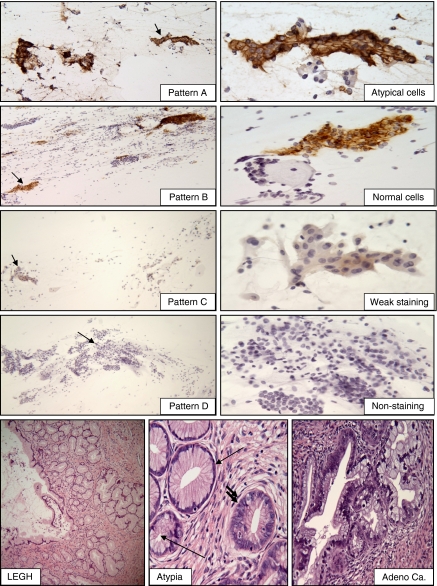
The four scoring patterns of CA-IX immunoreactivity in PSs containing AGC and representative illustrations of the cases with LEGH. Patterns **A** and **B**: positive immunostaining in the atypical cells/cell clusters (**A**) or in the normal looking endocervical cells (**B**). Patterns **C** and **D**: weak positive (**C**) or no immunoreactivity (**D**) in normal cervical cells. The original low power magnification ( × 100) of patterns **A**, **B**, **C** and **D** shown in the left panels and the corresponding cell clusters marked with arrow shown in the left panel ( × 400). The case of LEGH exhibits lobular proliferation of the small glands surrounding the larger gland (original magnification × 100) with focal nuclear atypia (single arrow: benign glands, double arrow: atypia) and the area of well-differentiated adenocarcinoma (original magnification, × 400).

CA-IX testing was performed in conventional study PS using the anti-CA-IX mouse monoclonal antibody, M75, as described previously (Liao *et al*, 1994; Liao and Stanbridge, 1996, 2000). Specific immunostaining was defined by the presence of a brown reaction product on the plasma membrane under × 40 magnification. Faint staining of the cytoplasm was considered negative. Cytological criteria for atypical cells, delineated in the Bethesda System classification, were used in the diagnostic classification (Kurman and Solomon, 1994). Immunostaining was scored as positive (pattern A and B) or negative (pattern C and D) based on the staining intensity (strong *vs* weak/negative) and immunoreactive patterns (diffuse *vs* focal). Strong positivity represented dark brown immunoreactivity that was easily identified at a low-power magnification ( × 4 or × 10). The diffuse staining pattern was defined as more than 50% of the cytologically atypical or normal endocervical cells in the smear showed immunoreactivity to CA-IX. The patterns A, B, C and D were defined as follows: (A) when individual atypical cells and/or cell clusters showed specific immunoreactivity that was either diffuse or focal; (B) when normal-looking endocervical cells exhibited focal or diffuse strong specific positive staining; (C) when the normal endocervical cells showed focal, but weak staining and; (D) when there was non-specific faint cytoplasmic positivity or lack of staining observed at × 40 magnification (Figure 1). A set of teaching smears with samples of known negative and positive CA-IX immunoreactivity was provided by one of the authors (SYL). After the training session, the CA-IX immunostained smears were evaluated and the interpretation in each case was recorded independently by 3 cyto/gynecologic pathologists (SYL, WHR and TAB) blinded to histological diagnosis. Any cases with different classification of immunostaining patterns were reviewed simultaneously by three study pathologists, using a multi-headed microscope. A consensus was obtained when at least two of the three study pathologists reached agreement. The results of the consensus were entered to the GOG Statistical database as the final score for each patient in the study.

### HPV genotyping

The detection method employed a modified E6/E7-specific consensus PCR, using mixed primers (pU-1M, pU-1M-L/pU-2R and pU-2R-N). The PCR method used was a minor modification of published procedures (Inoue *et al*, 1995; Yamazaki *et al*, 2001) in which modification of the primer sequences made it possible to amplify 13 H-HPV types (HPV 16, 18, 31, 33, 35, 39, 45, 51, 52, 58, 67, 68 and 70). The PCR-based procedure was performed on 84 of 88 specimens by the Takara Bio Corporation (Otsu, Shiga, Japan). The sequence of the PCR reactions was 94°C for 30 s, 55°C for 60 s and 72°C for 60 s, and each cycle was repeated 35 times. PCR product size ranged from 231 to 271 base-pairs and represent the E6 and E7 regions of HPV 16, 18, 31, 33, 35, 39, 45, 51, 52, 58, 67, 68 or 70. Genotyping of H-HPV DNA was performed according to the restriction fragment length polymorphism method described by Lungu *et al* (1992). In situations where genotyping was not detected by restriction enzyme analysis, genotyping was determined by direct sequencing of the amplified products.

### Statistical methods

Statistical analyses were performed using Statistical Analysis System version 9.1 (SAS Institute Inc., Cary, NC, USA). Sensitivity, specificity, positive predictive value and negative predictive value, interpreted as the risk of a SCL among women who test negative for H-HPV and/or CA-IX, and overall accuracy were calculated using the definition of FNR as 1 minus negative predictive value for women diagnosed by CA-IX or H-HPV status, individually or jointly, relative to histological diagnosis. When used in combination, the following decision rule was employed: if either CA-IX or HPV was positive, then the case was said to be test positive.

## Results

A total of 92 Japanese women with a cytological diagnosis of AGC were enrolled in the study. Four women were excluded because of the unsatisfactory study PS (*n*=3) and incomplete histological evaluation of the cervix (*n*=1). The age distribution of the patients ranged from 29 to 80, with a median age of 46. Details are given in Table 1. All patients were of Asian origin. Of the 88 eligible patients enrolled in the study, 37 (42%) women had a SCL. Among those with a SCL (*n*=37), 15 (41%) harboured a significant SL, including CIN2 (*n*=3), CIN3 (*n*=11) and squamous cell carcinoma (*n*=1), and 22 (59%) had a significant GL, comprising AIS (*n*=14), invasive adenocarcinoma (*n*=7) and adenosquamous carcinoma (*n*=1). Details are presented in Table 1.

### Accuracy of CA-IX testing

Of the 88 women enrolled in the study, 60 (68%) cases showed positive staining for the CA-IX in the conventional study PS. Among these positive cases, 33 (55%) had a SCL, including 11 SLs and 22 GLs. Thus, CA-IX immunoreactivity identified 33 of 37 (89%) SCLs with sensitivities of 73% for SLs and 100% for GLs. The overall specificity was 47%, with a FNR, defined as one minus the negative predictive value, equal to 14%, which indicates that 14% of the negative diagnoses were incorrect. Details are presented in Tables 2 and 3.

### Accuracy of H-HPV testing

The PCR-based H-HPV genotyping could only be performed in 84 of the eligible Japanese women with a liquid-cervical cell sample. H-HPV DNA was detected in 29 (35%) cases. Patient ages ranged from 29 to 71, with a median age of 43 for the H-HPV positive group and 49 for the H-HPV negative group (Table 1). Among the 29 H-HPV-positive cases, 22 (76%) had a SCL, including 12 SLs and 10 GLs. Thus, H-HPV testing identified 65% (22 out of 34) SCLs with sensitivities of 80% (12 out of 15) for SLs and 53% (10/19) for GLs. The overall specificity was 86%, with a FNR of 22% (Tables 2 and 3). The individual H-HPV genotyping results are given in Table 4. In the negative category, including glandular hyperplasia, atypia and CIN1, 7 of 50 (14%) specimens were positive. Each specimen had only one H-HPV type: three were type 52 and one each contained types 18, 33, 45 or 51. In the SL category, 12 of 15 (80%) specimens were positive. All of these positive specimens contained a single genotype: two were type 16; four were type 18; one was type 31; three were type 52; and one was type 58. The single squamous cell carcinoma specimen contained H-HPV types 16 and 33. Thus, the vast majority of SLs contained a single H-HPV genotype, with types 16, 18 and 52 being the most common. In the GLs, 10 of 19 (53%) were H-HPV positive. Of these 10 positive cases, two were type 16, seven were type 18 and 1 contained types 16 and 18. Details are presented in Tables 2 and 4.

### The combined accuracy of CA-IX and H-HPV testing

Combined CA-IX and H-HPV testing detected the additional 11 H-HPV-negative cases (two CIN3 and nine GLs). Thus, the overall sensitivity for SCLs, GLs or SLs was 97, 100 or 93%, respectively, with a specificity of 42%, and a FNR of 5% (Table 3).

### Retrospective histological evaluation

Retrospective histological evaluation was conducted on all cases by one of the authors (SYL). The diagnosis was confirmed in all cases but two. Those two cases were entered in the GOG database as CIN3 and adenosquamous carcinoma, respectively, but no SCLs could be identified retrospectively. Surprisingly, when the criteria proposed by Nucci *et al* for lobular endocervical glandular hyperplasia (LEGH) (Nucci *et al*, 1999) were applied to the pathological diagnoses, 13 cases with histological features of LEGHs were identified (Figure 1). All of these cases were independently reconfirmed by the second pathologist (WHR) blinded to the H-HPV and CA-IX data. The age distribution of cases with LEGH ranged from 36 to 80 with the mean of 54. Those cases with LEGHs were diagnosed as benign cervix (*n*=3), glandular hyperplasia (*n*=2), atypia (*n*=1), CIN2 (*n*=1) and GLs (*n*=6) by the panel of pathologists at the GOG central review. Six of 13 cases (43%) with LEGHs had coexistent *in-situ* (*n*=3) and/or extremely well-differentiated invasive adenocarcinoma, also termed ‘minimal deviation adenocarcinoma’ (*n*=3). All 13 LEGHs were HPV negative, but showed positive CA-IX expression in the PS. None of H-HPV-positive GLs had a coexistent LEGH histology. The results of retrospective histological evaluation and the corresponding H-HPV and CA-IX data are shown in Table 5.

## Discussion

In agreement with previous studies, a wide spectrum of benign and clinically significant lesions was identified in follow-up evaluation of patients with a diagnosis of AGC enrolled in the Japanese cohort of GOG protocol #171. The rate of significant uterine lesions in published studies of AGC has ranged from 17 to 80% (mean, 41%), with ranges of 0–34% (mean, 11%) for women with GLs, 5–43% (mean, 17%) for those with SLs and 0–23% (mean, 9%) for those with invasive carcinomas, with most of the latter being of endocervical or endometrial origin (Veljovich *et al*, 1998; Cangiarella and Chhieng, 2003). In this study we report that 42% (37/88) of women had a SCL, including 15 with a SL and 22 with a GL. The overall rate of invasive carcinoma was 10%. Among the 37 SCLs, 59% were GLs and 24% were invasive cervical carcinoma. The results are within the ranges formerly published.

H-HPV DNA detection has been strongly linked with SCLs; however, published data on the utility of H-HPV testing in AGC diagnosis is limited. Our recent data suggested that, in the United States, H-HPV testing is a useful biomarker for identifying SCLs in women with a cytological diagnosis of AGC, and has an overall sensitivity of 97, 100 or 96% for SCLs, GLs or SLs, respectively, and a FNR of 1% (Liao *et al*, 2009). The current study, however, revealed a low detection rate for H-HPV in SCLs with overall sensitivities of 65, 53 or 80% for SCLs, GLs or SLs, respectively, and a FNR of 22% in this cohort of Japanese women. Retrospective review of the histology of all H-HPV-negative GLs (*n*=8) identified six cases that were associated with LEGH. The diagnosis of LEGH in each specimen was confirmed by positive immunostaining of the lesion with an anti-MUC-6 antibody (Mikami *et al*, 2004, and data not shown). LEGH is a rare lesion of the uterine cervix; it usually occurs in postmenopausal women and has been proposed that LEGH represents pre-cancerous minimal deviation adenocarcinoma (extremely well-differentiated adenocarcinoma), independent of H-HPV infection.

None of the reported cases of LEGH contained H-HPV (Xu *et al*, 2005; Nara *et al*, 2007; Kawauchi *et al*, 2008). There were two cases of AIS without associated LEGH that were negative for H-HPV. In one AIS case the lesion was very focal and limited and the other AIS case stained negatively for p16, a biomarker strongly correlated with H-HPV expression (Negri *et al*, 2003). In contrast, high levels of p16 expression were seen in all H-HPV-positive SCLs (data not shown). Although one cannot exclude the possibility of a rare H-HPV type that was not represented in the testing kit, the findings appear to suggest that in this Japanese population, SCLs may not be solely due to H-HPV infection. Thus, H-HPV testing alone probably will not be able to identify all SCLs, especially in postmenopausal Japanese women. As mentioned in the results, there were two cases that were included in SCLs category for the data analysis; however, retrospective review of these two cases showed that there were no SCLs present in the tissue sections. These two cases were H-HPV negative. In order to avoid any bias issue the data presented in the paper were based on the results rendered by the central pathology review, which was blinded to the knowledge of histological diagnosis. However, if these two cases were reclassified as negative cases, there is a slightly increased sensitivity of H-HPV testing (69, 86 or 56% for SCLs, GLs or SLs, respectively).

Our current study confirms the diagnostic utility of CA-IX in detecting glandular neoplasia of the cervix (Liao *et al*, 1994; Liao and Stanbridge, 1996, 2000). Practically, all exfoliative endocervical glandular cells derived from the neoplastic glands in all cases of AIS and adenocarcinoma showed diffuse and strong CA-IX immunoreactivity. These CA-IX-positive cells are easily identified at × 4–10 magnification. The patterns of CA-IX expression are identical to those previously reported (Liao *et al*, 1994; Liao and Stanbridge, 2000; Liao *et al*, 2009). The high levels of CA-IX expression in LEGHs without associated GLs seen in the study is an intriguing finding; however, it has been proposed that LEGH may be a precursor of minimal deviation adenocarcinoma thus, further investigation is warranted.

Despite the relatively low detection rate of CA-IX for SLs, and H-HPV for GLs, the combined CA-IX/H-HPV testing had a sensitivity of 97, 93 and 100% for SCLs, SLs and GLs, with a FNR of 0.05%. If the results of retrospective review were used for data analysis then the combined CA-IX/H-HPV testing had a sensitivity of 100% for SCLs with 0% FNR. Thus, the study concludes that the combination of CA-IX with H-HPV testing significantly improved the diagnostic accuracy in Japanese women with AGC diagnosis; the low rate of H-HPV positivity in the GLs was probably attributed to coexisting LEGH independent of H-HPV; CA-IX testing identified all cases of GLs associated with LEGH; and lastly, most H-HPV-positive specimens contained a single genotype. The replacement protocol, GOG #237, opened to accrual in February 2009 to prospectively test the conclusions reported herein.

## Figures and Tables

**Table 1:** Clinical characteristics and histology diagnosis

**Clinical characteristics**			
**Patient age**			
	***n*=88 (%)** a	***n*=84 (%)** b	
		**HPV (PCR)**	
**Age**	**All cases**	**HPV (+)**	**HPV (−)**
⩽30	1 (1)	1 (1)	0
31–40	22 (25)	10 (12)	10 (12)
41–50	31 (35)	11 (13)	18 (23)
51–60	24 (27)	7 (8)	17 (20)
61–70	6 (7)	0	5 (6)
⩾71	4 (5)	0	4 (5)
			

**Histological diagnosis *n*=88 (%)** a					
**Insignificant cervical lesions 51 (58)**		**Significant cervical lesions 37 (42)**			
*Negative/benign*	34	*Squamous lesions*	15 (41)	*Glandular Lesions*	22 (59)
Negative, NOS^a^	24	CIN2	3	Adenocarcinoma *in situ*	14
Metaplasia, NOS	3	CIN3	11	Invasive adenocarcinoma	8
Squamous metaplasia	7	Squamous cell carcinoma	1	Adenocarcinoma NOS	7
Atypia^c^	6			Adenosquamous	1
Glandular hyperplasia	5				
CIN1: mild dysplasia	6				

Abbreviations: AIS=adenocarcinoma *in situ*; CA-IX=carbonic anhydrase IX; CIN=cervical intraepithelial neoplasia; HPV=human papillomavirus; NOS=not otherwise specified.

CIN2, moderate dysplasia; CIN3, severe dysplasia/*in situ* squamous carcinoma.

a A total of 88 cases were tested for CA-IX expression.

b Among 88 cases, 84 were also tested for high-risk HPV.

c Including glandular and squamous lesions in which cellular atypia falls short of AIS and CIN1.

**Table 2:** Biomarker test (CA-IX, HPV and CA-IX+HPV) results by histological diagnosis

	**Histological diagnosis**				
**Biomarker test**		**Insignificant cervical lesions**	**SLs**	**GLs**	**All SCLs (SLs+GLs)**
Total number^a^	88 (%)	51 (58)	15 (17)	22 (25)	37 (42)
					
*CA-IX*					
Negative	28 (32)	24 (47)	4 (27)	0	4 (11)
Positive	60 (68)	27 (53)^b^	11 (73)	22 (100)	33 (89)
					
Total number^c^	84 (%)	50 (59)	15 (18)	19 (23)	34 (40)
					
*CA-IX*					
Negative	47 (56)	43 (86)	4 (27)	0	4 (12)
Positive	37 (44)	7 (14)	11 (73)	19 (100)	30 (88)
					
*HPV (PCR)*					
Negative	55 (55)	43 (51)	3 (20)	9 (47)	12 (35)
Positive	29 (35)	7 (14)^b^	12 (80)	10 (53)	22 (65)
					
*CA-IX*+*HPV*					
Negative	22 (26)	21 (42)	1 (7)	0	1 (3)
Positive	62 (74)	29 (58)	14 (93)	19 (100)	33 (97)

Abbreviations: AIS=adenocarcinoma *in situ*; CA-IX=carbonic anhydrase IX; CIN=cervical intraepithelial neoplasia; GL=glandular lesion; HPV=human papillomavirus; SCL=significant cervical lesion; SL=squamous lesion.

Insignificant cervical lesions, including negative/benign, CIN1, atypia and glandular hyperplasia; SLs: CIN2, CIN3 and squamous cell carcinoma; GLs: AIS and adenocarcinoma.

a All cases tested for CA-IX.

b Including two CIN1, one atypia and one hyperplasia.

c Cases in which HPV (HC2) results were available.

**Table 3:** Diagnostic accuracy of CA-IX, HPV and CA-IX+HPV

		**Sensitivity**						
**Numbers tested**	**Biomarkers**	**SCLs**	**SLs**	**GLs**	**Specificity**	**NPV**	**PPV**	**FNR** a
88	CA-IX	0.89	0.73	1.00	0.47	0.86	0.55	0.14
84	CA-IX	0.88	0.73	1.00	0.50	0.86	0.54	0.14
	HPV (PCR)	0.65	0.80	0.53	0.86	0.78	0.76	0.22
	HPV (PCR)+CA-IX	0.97	0.93	1.00	0.42	0.95	0.53	0.05

Abbreviations: CA-IX=carbonic anhydrase IX; FNR=false negative rate; GL=glandular lesion; HPV=human papillomavirus; NPV=negative predictive value; PPV=positive predictive value; SCL=significant cervical lesion; SL=squamous lesion

a FNR: defined as 1 NPV to reflect the proportion of negative diagnosis that was incorrect.

**Table 4:** Distribution of HPV types in individual HPV-positive cases

		**HPV genotypes**											
**Histological diagnosis**	**Number of HPV-positive cases**	**16**	**18**	**31**	**33**	**35**	**39**	**45**	**51**	**52**	**58**	**67**	**70**
Negative/benign	4				1			1		2			
Glandular hyperplasia	0												
Atypia	0												
CIN1 (mild dysplasia)	3		1						1	1			
CIN2	2		1	1									
CIN3	9	2	3							3	1		
Squamous cell carcinoma^a^	1	1			1								
Adenocarcinoma *in situ*^b^	6	3	4										
Adenocarcinoma, NOS	4		4										
Total HPV-positive cases	29	6	13	1	2			1	1	6	1		

Abbreviations: CIN=cervical intraepithelial neoplasia; HPV=human papillomavirus; NOS=not otherwise specified.

CIN2: moderate dysplasia; CIN3: severe dysplasia/*in situ* squamous carcinoma. Note: one squamous cell carcinoma.

a Contained HPV 16 and 31 and one adenocarcinoma *in situ*.

b Contained HPV 16 and 18; all other positive cases contained a single HPV type.

**Table 5:** Retrospective histological evaluation with the correlation of HPV status and CA-IX expression in the Pap smears

**Histology**	**Case no. (*N*=84)**	**HPV no. of positive cases (%)**	**CA-IX expression no. of positive cases (%)**
Negative/benign	32	4 (13)	12 (39)
Glandular hyperplasia NOS	3	0 (0)	0 (0)
Atypia	5	0 (0)	2 (40)
CIN1	6	3 (50)	5 (83)
			
*SCLs without LEGH*			
CIN 2, CIN 3	12	11(92)	10 (83)
AIS	8	6 (75)^a^	8 (100)
Adenocarcinoma	4	4 (100)	4 (100)
Squamous cell carcinoma	1	1 (100)	1 (100)
LEGH	6	0 (0)	6 (100)
			
*LEGH with SCLs*			
CIN 2	1	0 (0)	1 (100)
AIS	3	0 (0)	3 (100)
Adenocarcinoma	3	0 (0)	3 (100)

Abbreviations: AIS=adenocarcinoma *in situ*; CA-IX=carbonic anhydrase IX; CIN=cervical intraepithelial neoplasia; HPV=human papillomavirus; LEGH=lobular endocervical glandular hyperplasia; NOS=not otherwise specified; SCLs=significant cervical lesions.

a Two HPV-negative cases, in one case was also p16 negative and in the other the lesion was limited.

## References

[bib1] Andersen ES, Arffmann E (1989) Adenocarcinoma *in situ* of the uterine cervix. A clinico-pathologic study of 36 cases. Gynecol Oncol 35: 1–7 2792895 10.1016/0090-8258(89)90001-2

[bib2] Bosch FX, Burchell AN, Schiffman M, Giuliano AR, de Sanjose S, Bruni L, Tortolero-Luna G, Kjaer SK, Muñoz N (2008) Epidemiology and natural history of human papillomavirus infections and type specific implications in cervical neoplasia. Vaccine 26(Suppl 10): K1–K16 18847553 10.1016/j.vaccine.2008.05.064

[bib3] Bray F, Carstensen B, Møller H, Zappa M, Žakelj MP, Lawrence G, Hakama M, Weiderpass E (2005) Incidence trends of adenocarcinoma of the cervix in 13 European countries. Cancer Epidemiol Biomarkers Prev 14: 2191–2199 16172231 10.1158/1055-9965.EPI-05-0231

[bib4] Cangiarella JF, Chhieng DC (2003) Atypical glandular cells-an update. Diagn Cytopathol 29: 271–279 14595795 10.1002/dc.10316

[bib5] Inoue Y, Yamashita T, Ishida S, Nishikawa A, Fujinaga Y, Kudo R, Fujinaga K (1995) Detection and typing of genital high-risk HPV DNAs in cervical scrapes using the E6/E7-specific consensus PCR. Tumor Res 30: 1–19

[bib6] Ivanov S, Liao SY, Ivanova A, Danilkovitch-Miaqkova A, Tarasova N, Weirich G, Merrill MJ, Proescholdt MA, Oldfield EH, Lee J, Zavada J, Waheed A, Sly W, Lerman MI, Stanbridge EJ (2001) Expression of hypoxia-inducible cell-surface transmembrane carbonic anhydrases in human cancer. Am J Pathol 158: 905–919 11238039 10.1016/S0002-9440(10)64038-2 PMC1850340

[bib7] Kawauchi S, Kusuda T, Liu XP, Suehiro Y, Kaku T, Mikami Y, Takeshita M, Nakao M, Chochi Y, Sasaki K (2008) Is lobular endocervical glandular hyperplasia a cancerous precursor of minimal deviation adenocarcinoma? A comparative molecular-genetic and immunohistochemical study. Am J Surg Pathol 32: 1807–1815 18779726 10.1097/PAS.0b013e3181883722

[bib8] Kurman RJ, Solomon D (1994) The Bethesda System: Terminology for Reporting Cervical/Vaginal Cytologic Diagnoses: Definitions, Criteria and Explanatory Notes for Terminology and Specimen Adequacy. Springer Verlag: New York

[bib9] Lee KR, Darragh TM, Joste NE, Krane JF, Sherman ME, Hurley LB, Allred EM, Manos MM (2002) Atypical glandular cells of undetermined significance (AGUS): interobserver reproducibility in cervical smears and corresponding thin-layer preparations. Am J Clin Pathol 117: 96–102 11789738 10.1309/HL0B-C7Y6-AC77-ND2U

[bib10] Liao SY, Aurelio ON, Jan K, Závada J, Stanbridge EJ (1997) Identification of the MN/CA9 protein as a reliable diagnostic biomarker of clear cell carcinoma of the kidney. Cancer Res 57: 2827–2831 9230182

[bib11] Liao SY, Brewer C, Závada J, Pastorek J, Pastorekova S, Manetta A, Berman ML, DiSaia PJ, Stanbridge EJ (1994) Identification of the MN antigen as a diagnostic biomarker of cervical intraepithelial squamous and glandular neoplasia and cervical carcinoma. Am J Pathol 145: 598–609 8080042 PMC1890321

[bib12] Liao SY, Rodgers WH, Kauderer J, Bonfiglio TA, Walker JL, Darcy KM, Carter R, Hatae M, Levine L, Spirtos NM, Stanbridge EJ (2009) Carbonic anhydrase IX (CA-IX) and human papillomavirus (HPV) as diagnostic biomarkers of cervical dysplasia/neoplasia in women with a cytologic diagnosis of atypical glandular cells (AGC): a Gynecologic Oncology Group (GOG) Study. Int J Cancer 25: 2434–2440 10.1002/ijc.24615 PMC2779726 19670419

[bib13] Liao SY, Stanbridge EJ (1996) Expression of the MN antigen in cervical Papanicolaou smears is an early diagnostic biomarker of cervical dysplasia. Cancer Epidemiol Biomarkers Prev 5: 549–557 8827360

[bib14] Liao SY, Stanbridge EJ (2000) Expression of MN/CA9 protein in Papanicolaou smears containing atypical glandular cells of undetermined significance is a diagnostic biomarker of cervical dysplasia and neoplasia. Cancer 88: 1108–1121 10699902 10.1002/(sici)1097-0142(20000301)88:5<1108::aid-cncr23>3.0.co;2-d

[bib15] Lungu O, Wright Jr TC, Silverstein S (1992) Typing of human papillomaviruses by polymerase chain reaction amplification with L1 consensus primers and RFLP analysis. Mol Cell Probes 6: 145–152 1355266 10.1016/0890-8508(92)90059-7

[bib16] Makino H, Sato S, Yajima A, Komatsu S, Fukao A (1995) Evaluation of the effectiveness of cervical cancer screening: a case-control study in Miyagi, Japan. Tohoku. J Exp Med 175: 171–178 10.1620/tjem.175.171 7792786

[bib17] Mikami Y, Kiyokawa T, Hata S, Fujiwara K, Moriya T, Sasano H, Manabe T, Akahira J-I, Ito K, Tase T, Yaegashi N, Sato I, Tateno H, Naganuma H (2004) Gastrointestinal immunophenotype in adenocarcinomas of the uterine cervix and related glandular lesions: a possible link between lobular endocervical glandular hyperplasia/pyloric gland metaplasia and ‘adenoma malignum’. Modern Pathol 17: 962–972 10.1038/modpathol.3800148 15143335

[bib18] Nara M, Hashi A, Murata SI, Kondo T, Yuminnamochi T, Nakazawa K, Katoh R, Hoshi K (2007) Lobular endocervical glandular hyperplasia as a precursor of cervical adenocarcinoma independent of human papillomavirus infection. Gynecol Oncol 106: 289–298 17540439 10.1016/j.ygyno.2007.03.044

[bib19] Negri G, Egarter-Vigl E, Kasal A, Romano F, Haitel A, Mian C (2003) p16^INK4a^ is a useful marker for the diagnosis of adenocarcinoma of the cervix uteri and its precursors: an immunohistochemical study with immunocytochemical correlations. Am J Surg Pathol 27: 187–193 12548164 10.1097/00000478-200302000-00006

[bib20] Nucci MR, Clement PB, Young RH (1999) Lobular endocervical glandular hyperplasia, not otherwise specified: a clinicopathologic analysis of thirteen cases of a distinctive pseudoneoplastic lesion and comparison with fourteen cases of adenoma malignum. Am J Surg Pathol 23: 886–891 10435557 10.1097/00000478-199908000-00005

[bib21] Opavsky R, Pastoreková S, Zeinik V, Gibadulinová A, Stanbridge EJ, Závada J, Kettmann R, Pastorek J (1996) Human *MN/CA9 gene,* a novel member of the carbonc anhydrase family: structure and exon to protein domain relationships. Genomics 33: 480–487 8661007 10.1006/geno.1996.0223

[bib22] Pagliusi S (2006) World health Organization. Human papillomavirus infection and cervical cancer. Available at: http://www.who.int/vaccine_research/diseases/hpv/en/

[bib23] Pirog EC, Kleter B, Olgac S, Bobkiewicz P, Lindeman J, Quint WG, Richart RM, Isacson C (2000) Prevalence of human papillomavirus DNA in different histological subtypes of cervical adenocarcinoma. Am J Pathol 157: 1055–1062 11021808 10.1016/S0002-9440(10)64619-6 PMC1850168

[bib24] Schiffman M, Castle PE, Jeronimo J, Rodriquez AC, Wacholder S (2007) Human papilloma virus and cervical cancer. Lancet 370: 890–907 17826171 10.1016/S0140-6736(07)61416-0

[bib25] Smith HO, Tiffany MF, Qualls CR, Key CR (2000) The rising incidence of adenocarcinoma relative to squamous cell carcinoma of the uterine cervix in the United States-a 24-year population-based study. Gyneco Oncol 78: 97–105 10.1006/gyno.2000.5826 10926787

[bib26] Solomon D, Davey D, Kurman R, Moriarty A, O’Connor D, Prey M, Raab S, Sherman M, Wilbur D, Wright Jr T, Young N (2002) Forum Group Members; Bethesda 2001 Workshop. The 2001 Bethesda system- Terminology for reporting results of cervical cytology. JAMA 287: 2114–2119 11966386 10.1001/jama.287.16.2114

[bib27] Solomon D, Schiffman M, Tarone R (2001) Comparison of three management strategies for patients with atypical squamous cells of undetermined significance: baseline results from a randomized trial. J Natl Cancer Inst 93: 293–299 11181776 10.1093/jnci/93.4.293

[bib28] Swietach P, Vaughan-Jones RD, Harris AL (2007) Regulation of tumor pH and the role Of carbonic anhydrase 9. Cancer Metastasis Rev 26: 299–310 17415526 10.1007/s10555-007-9064-0

[bib29] Veljovich DS, Stoler MH, Andersen WA, Covell JL, Rice LW (1998) Atypical glandular cells of undetermined significance: a five-year retrospective histopathologic study. Am J Obstet Gynecol 179: 382–390 9731842 10.1016/s0002-9378(98)70368-0

[bib30] Wykoff CC, Beasley NJ, Watson PH, Turner KJ, Pastorek J, Sibtain A, Wilson GD, Turley H, Talks KL, Maxwell PH, Pugh CW, Ratcliffe PJ, Harris AL (2000) Hypoxia- Inducible expression of tumor-associated carbonic anhydrases. Cancer Res 60: 7075–7083 11156414

[bib31] Xu JY, Hashi A, Kondo T, Yuminamochi T, Nara M, Murata S, Katoh R, Hoshi K (2005) Absence of human papillomavirus infection in minimal deviation adenocarcinoma and lobular endocervical glandular hyperplasia. Int J Gynecol Pathol 24: 296–302 15968208 10.1097/01.pgp.0000157918.36354.c1

[bib32] Yamazaki H, Sasagawa T, Basha W, Segawa T, Inoue M (2001) Hybrid capture II and LCR-E7 PCR assays for HPV typing in cervical cytologic samples. Int J Cancer 94: 222–227 11668502 10.1002/ijc.1455

[bib33] Zavada J, Zavadova Z, Pastorekova S, Ciampor F, Pastorek J, Zelnik V (1993) Expression of MaTu-MN protein in human tumor cultures and in clinical specimens. Int J Cancer 54: 268–274 8486430 10.1002/ijc.2910540218

[bib34] zur Hausen H (2002) Papillomaviruses and cancer: from basic studies to clinical application. Nat Rev Cancer 2: 342–350 12044010 10.1038/nrc798

